# Interstitial Lung Disease-Complicated Anti-MDA5 Antibody in Clinically Amyopathic Dermatomyositis Patients: Report of Two Cases With Distinct Clinical Features

**DOI:** 10.3389/fmed.2020.00077

**Published:** 2020-03-10

**Authors:** Laurence Pacot, Jacques Pouchot, Nicolas De Prost, Marie Senant, Eric Tartour, Françoise Le Pimpec-Barthes, Dominique Israel-Biet, Marie-Agnes Dragon-Durey

**Affiliations:** ^1^Service d'Immunologie Biologique, Hôpital Européen Georges Pompidou, Paris, France; ^2^Service de Médecine Interne, Hôpital Européen Georges Pompidou, Paris, France; ^3^Service de Réanimation Médicale, Hôpital Henri Mondor, Créteil, France; ^4^Université Paris-Descartes, Paris, France; ^5^Service de Chirurgie Thoracique, Hôpital Européen Georges Pompidou, Paris, France; ^6^Service de Pneumologie, Hôpital Européen Georges Pompidou, Paris, France

**Keywords:** autoantibodies, dermatomyositis, skin rash, interstitial lung disease, anti-rejection therapy, lung transplantation, anti-MDA5

## Abstract

Two patients presented simultaneously to our hospital with distinct clinical features associated with the presence of anti-MDA5 antibodies: the first one was admitted for a skin rash resembling to a toxic epidermal necrosis (Lyell syndrome) and the second one presented with pulmonary manifestations attributed to a diffuse fibrosing interstitial pneumonitis on chest CT-scan. In addition to the skin lesions involving 40% of the body surface area, the first patient developed a rapid diffuse interstitial pneumonitis with respiratory distress justifying the initiation of a systemic immunosuppressive treatment. However, she died 3 weeks after her admission from mesenteric thrombosis associated with septic shock. The second patient respiratory condition worsened despite an intensive immunosuppressive treatment with high doses of intravenous methylprednisolone and cyclophosphamide and plasmapheresis, and required lung transplantation. Anti-MDA5 antibody titer declined and disappeared on anti-rejection treatment. These two cases underline the diagnostic conundrum and the therapeutic difficulties in patients with anti-MDA5 antibodies and clinically amyopathic dermatomyositis (CADM) or interstitial lung disease (ILD), who may undergo rapidly-progressive and fatal outcome. Presence of anti-MDA5 antibodies should always be suspected when confronted to CADM patients with cutaneous ulcerations or ILD to allow a rapid and adapted treatment initiation.

## Background

Polymyositis/dermatomyositis (PM/DM) are systemic inflammatory myopathies which involve skeletal muscles, skin and possibly other organs like joints and lung. Annual incidence of PM/DM is ~8.5 per million (1). In its clinically amyopathic form (CADM), which represents ~20% of all DM (2), the disease is mostly characterized by cutaneous lesions such as ulcerations, palm papules or Gottron's sign that persist for more than 6 months without sign of muscle weakness (Sontheimer criteria). Incidence of PM/DM-related interstitial lung diseases (ILD) ranges from 5 to 65%, depending on whether clinical, radiological, functional or histological criteria are used (3). Among these patients the presence of anti-synthetase antibodies seems to be of a relatively good prognosis (4, 5) whereas mortality rates can be high in other autoimmune-related ILD, with a poor response to immunosuppressive therapy, particularly in CADM-ILD. In 2005, Sato and collaborators described for the first time an antibody recognizing a 140 kDa protein (anti-CADM-140 antibodies) which was associated with CADM in Asian patients, who tended to develop rapidly-progressive ILD (RP-ILD) (6). They later on identified this 140 kDa protein as being melanoma differentiation–associated gene 5 (MDA5) (7), a protein implicated in long double-strand RNA recognition, notably picornaviruses RNAs, that activates the interferon signaling pathway through the adaptor molecule MAVS (8).

Since then, many studies have confirmed that anti-MDA5 antibodies are most frequently detected in CADM patients, representing up to 50–73% of CADM (2), and are often associated with ILD (9). Moreover, skin ulcerations are more frequent and severe in anti-MDA5 positive patients than in DM/CADM patients without anti-MDA5 antibodies (10).

We here report on two Caucasian patients, positive for anti-MDA5 antibodies, who were simultaneously admitted to the same hospital, with distinct clinical features.

## Case Presentation

### Case #1

**Figure 1:**
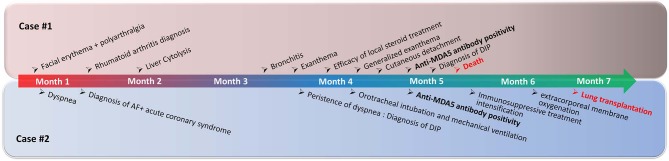
Chronological representation of the main clinical data of Case #1 **(top)** and Case #2 **(bottom)**. AF, Atrial fibrillation; DIP, diffuse interstitial pneumonitis.

A 59-year-old woman presenting with facial erythema and polyarthralgia was diagnosed with rheumatoid arthritis and treated by methotrexate (Figure 1). She rapidly developed hepatic cytolysis that persisted after a switch to hydroxychloroquine. Three months later, she developed vesicles and pustules on the shoulders, which firstly responded to a local corticosteroid treatment. The appearance of large cutaneous erosions on the back, chest, arms and the legs and necrotic skin lesions over the elbows and the ankles one month later led to suspect a Lyell syndrome, justifying her admission in the hospital. Cutaneous lesions of this patient have previously been described in a short letter (11). Her past medical history included a splenectomy for idiopathic thrombocytopenic purpura at the age of 37 years, an allergic asthma and a nasal polyposis.

**Figure 2:**
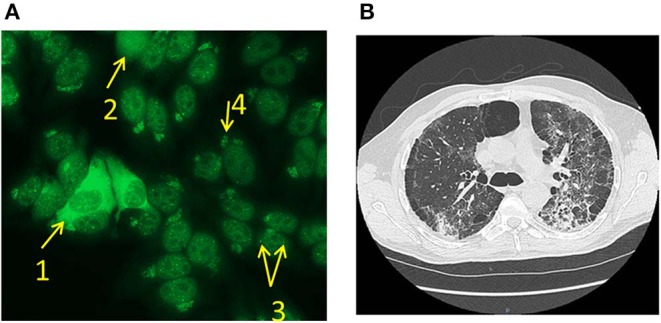
**(A)** ANA aspect of Case #1. IIF on Hep-2 (1/80) revealed presence of rare isolated cytoplasmic islets (1), homogenous staining (2), multiple nuclear dots pattern (3) and anti-Golgi apparatus pattern (4). **(B)** Radiographic imaging of Case #2. Thoracic computed tomography scan revealed bilateral interstitial lung disease with lower lung predominance, thickened alveolar septa, condensations, and traction bronchiectasis.

At the time of diagnosis, the patient showed anemia and moderate hepatic cytolysis (Table 1). Presence of antinuclear antibodies (ANA) was revealed by indirect immunofluorescence (IIF) on Hep-2 cells (Euroimmun©, Germany) with the association of a homogenous staining, multiple nuclear dots pattern, anti-Golgi apparatus pattern, and rare isolated cytoplasmic islets positivity (Figure 2). Antigenic specificity was studied by immunodots (Euroimmun©, Autoimmune Inflammatory Myopathies and Autoimmune Hepatopathies, Germany) using the systems EUROBlotMaster and EUROLineScan (Euroimmun©, Germany), and revealed high levels of anti-MDA5 (with an intensity of the antigen band superior to 10-fold the positive threshold), anti-SSA 52 (Trim 21) and anti-Sp-100 autoantibodies.

**Table 1:** Biological findings at diagnosis.

	**Hb** **(Females 12–16 g/dL; males 13–17g/dL)**	**Platelets** **(150–450G/L)**	**WBC** **(4–10G/L)**	**Creatininemia** **(<80 μmol/L)**	**ALP** **(35–105 U/L)**
Case #1	**8.3**	425	7,9	52	**156**
Case #2	**11.9**	333	**11**	47	82
	**AST** **(<35 U/L)**	**ALT** **(<35 U/L)**	**GGT** **(<40 U/L)**	**Bilirubin** **(<21** **μmol/L)**	**LDH** **(<250 U/L)**
Case #1	**52**	21	**239**	11	**461**
Case #2	27	**43**	**97**	9	**348**

*Normal ranges are indicated into brackets. Abnormal values are in bold characters. ALP, alkaline phosphatase; ALT, alanine transaminase; AST, aspartate transaminase; GGT, gamma-glutamyltransferase; Hb, hemoglobin; LDH, lactate dehydrogenase; WBC, white blood cells*.

During the time of her hospitalization, the cutaneous erosions spread rapidly and reached 40% of the body surface. Because of a concomitant respiratory distress, a systemic immunosuppressive treatment with 500 mg/day of methylprednisolone for 3 days was initiated, followed by 1.5 mg/kg of prednisone per day and one pulse of 0.6 g/m^2^ of cyclophosphamide. Despite this treatment, a diffuse interstitial pneumonitis rapidly developed, followed by a mesenteric thrombosis with multiple organ failure and a septic shock. The patient ultimately died from a systemic infection, 3 weeks only after diagnosis.

### Case #2

A 51-year-old man presented with dyspnea associated with atrial fibrillation and an acute coronary syndrome treated by angioplasty (Figure 1). His past medical history revealed a 45 pack-year smoking and dyslipidemia. As the dyspnea persisted, a lung CT-scan showed a diffuse fibrosing interstitial pneumonitis (Figure 2). Immunological analysis revealed only very rare cytoplasmic islets positivity in IIF and anti-MDA5 associated with anti-SSA 52 antibodies were detected.

**Figure 3:**
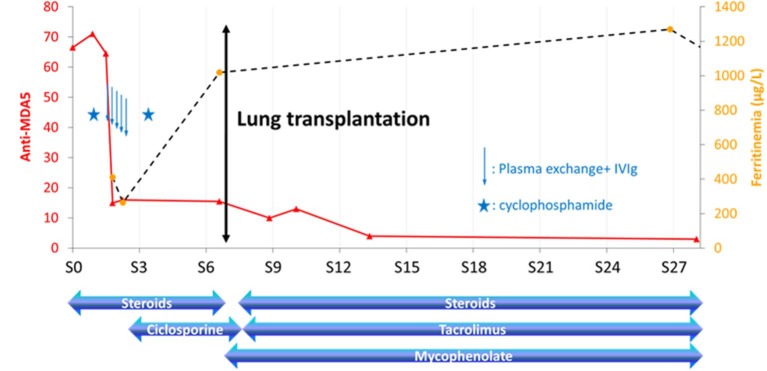
Biological follow-up and treatment of Case #2. The full curve represents the regression of anti-MDA5 antibodies titers (expressed as relative intensity) and the dotted curve the evolution of serum ferritin level. Five plasma exchanges were performed (arrows), preceded and followed by cyclophosphamide infusions (depicted with stars). MDA5, melanoma differentiation–associated gene 5.

After 5 days of bolus corticosteroids (500 mg) with only modest impact on respiratory distress, he received a first pulse of intravenous cyclophosphamide (600 mg/m^2)^ associated with 1 mg/kg of oral corticosteroids then, 3 days afterwards, five sessions of plasmapheresis combined with 4 mg/kg/d of ciclosporin. A second pulse of cyclophosphamide was performed at D25 with a continued treatment with 400 mg/d of ciclosporin and 20 mg/d of corticosteroids. Respiratory failure required an extracorporeal membrane oxygenation (ECMO) as a bridge to lung transplantation which was performed 7 weeks after diagnosis. Post-transplantation treatment included 1,500 mg of mycophenolate and 11 mg of tacrolimus twice a day, together with 1,000 mg of intravenous methylprednisolone. Since transplantation, the immunological monitoring has showed no reappearance of anti-MDA5 antibodies after 20 weeks of antirejection treatment and a slow normalization of ferritinemia, measured on DxI800 (Beckman-Coulter©, France), was observed (Figure 3). Three years after lung transplantation, the patient is alive.

## Discussion

The cutaneous manifestations of dermatomyositis and clinically amyopathic dermatomyositis include Gottron papules, Gottron sign, which are almost pathognomonic of these pathologies, periorbital heliotrope erythema, periungual telangiectasias and photodistributed erythema or poikiloderma (12). The occurrence of skin ulcerations, which affect 3–19% of DM patients (13), is more frequently observed in CADM patients than in DM patients (12), especially when associated with anti-MDA5 antibodies (13, 14). In this latter form of CADM, cutaneous ulcerations may overlie Gottron papules over the digital pulp and periungual areas or Gottron sign over the elbows and knees, and may more generally develop on sun-exposed sites, such as chest, back, and arms. Vasculopathy may be a major actor in the development of such ulcerations (10). The other classical clinical features of anti-MDA5 positive patients include palmar papules, alopecia, painful oral ulcers, panniculitis, mechanic hands, arthritis, arthralgias, fever, and ILD (10, 13, 14). Biological findings may include lymphopenia and elevated levels of ferritin, erythrocyte sedimentation rate and interleukin 18 (12, 14) with no increase in serum creatinine kinase (CK). Our first patient indeed presented with fever and arthralgias and developed oropharyngeal ulcerations and ulcerative and necrotic lesions over the limbs with large skin erosions before the diagnosis of diffuse interstitial pneumonitis. Furthermore, she died of septic shock following mesenteric thrombosis, and polymyositis/dermatomyositis patients have an increased risk of venous thromboembolism (15). The mechanisms underlying this pro-thrombotic condition are still largely unknown but may be at least partially explained by a hypercoagulability state due to systemic inflammation in PM/DM patients (16). Concerning CK dosage, the first patient had a transient increase up to 4,100 U/L with an electromyogram compatible with a myogenic syndrome. Case #2 had normal CK levels.

ILD is frequently observed early in the disease course of PM/DM patients when using imaging even though respiratory symptoms may be absent (1). Its prognosis is particularly severe in CADM-ILD associated with anti-MDA5 antibodies (4, 5, 7) where it often progresses toward a rapidly progressive fibrosing ILD leading to respiratory failure despite intensive immunosuppression. Only few cases of lung transplantation have been reported in the context of dermatomyositis, often regarded as a contra-indication in these patients. Shoji et al. reported the case of a female patient with a rapidly progressive ILD associated with anti-MDA-5 antibodies that underwent successful lung transplantation after an intensive immunosuppressive therapy including cyclophosphamide and intravenous immunoglobulins (17). Other similar cases have been reported since then, with favorable outcomes (18, 19). Like our two patients, at least one of them had anti-SSA 52 antibodies (18), which are frequently associated with anti-MDA5 antibodies (10) and seem to be correlated with severe forms of ILD (2, 20). As illustrated by our patient #2, some cases may present with RP-ILD and poor or no cutaneous, articular or muscle involvement (2, 21), making diagnosis even more tricky and may delay proper support.

Some biological parameters have been evaluated as potential predictive markers of outcome in anti-MDA5 positive patients. Serum ferritin level has been proposed as a predictor of RP-ILD (when ≥217 ng/mL) and fatal outcome (when ≥828 ng/mL) in these patients (22). This study also revealed that anti-MDA5 antibody titers and IL-18 levels decreased significantly after treatment in the RP-ILD patients who survived, meaning that sustained high levels or reappearance of these parameters represent potential biomarkers of unfavorable outcome or relapse in RP-ILD patients with positive anti-MDA5 antibodies (23). Anti-MDA5 antibody levels are also correlated to the severity of skin ulcerations and ILD, as well as with acute/subacute vs. chronic form of the lung disease, with a cutoff at 500 units/mL (9). However, this threshold has to be calculated for each anti-MDA5 assay due to a lack of standardization. The detection of anti-MDA5 antibodies among DM-associated ILD might be a keystone to anticipate resistance to treatment and survival, as anti-MDA5-positive patients who exhibit initial response to treatment achieve long-term survival (4).

There is currently no consensus on the best treatment of anti-MDA5 positive patients with RP-ILD. Treatments targeting B-cell functions, like the chimeric monoclonal anti-CD20 antibody rituximab, have shown some efficiency by reducing the production of anti-MDA5 antibodies (24). However, such molecules considerably increase the infectious risk. On the other hand, plasma exchanges represent a reasonable alternative for refractory cases, though adverse events such as allergy cannot be predicted (25). Altogether, combined immunosuppressive therapies, possibly by adjunction of mycophenolate and/or tacrolimus, give promising results (26). Large prospective studies are still lacking to identify the optimal treatment in anti-MDA5 positive patients with RP-ILD, but the scarcity of such patients will most likely require the set-up of multicenter clinical trials.

In the two patients presented here, the diagnostic delay was about 4 months. Because of its unique clinical presentation and very discrete aspect in IIF, most often described as negative in ANA (10), anti-MDA5-associated-DM is still a diagnostic challenge and its prevalence is probably underestimated. Patients often exhibit mild to absent increase of muscle enzymes, concordant with the amyopathic form of the disease.

The two cases described here exemplify the difficulties of diagnosing and treating patients with anti-MDA5 positive DM or ILD, who may undergo rapidly-progressive and fatal outcome. Anti-MDA5 antibodies must be suspected in CADM patients with cutaneous ulcerations or ILD to allow rapid treatment initiation.

## Ethics Statement

This study was performed in accordance with the Helsinki Declaration as revised in 2013 and Good Clinical Practice guidelines. It has been approved by the Institutional Review Board according to standards currently applied in France (Commission Nationale de l'Informatique et des Libertés, CNIL N°1922081 from 02/02/2016). Written informed consent was obtained from the participants or from their relatives for the use of their social and medical data for publication of this case report.

## Author Contributions

LP, MS, ET, and M-AD-D performed the biological analysis and wrote the manuscript. JP, ND, FL, and DI-B took care of the patients and wrote the manuscript.

### Conflict of Interest

The authors declare that the research was conducted in the absence of any commercial or financial relationships that could be construed as a potential conflict of interest.
